# Rearrangement hotspots in the sex chromosome of the Palearctic black fly *Simulium bergi* (Diptera, Simuliidae)

**DOI:** 10.3897/CompCytogen.v10i2.8855

**Published:** 2016-06-30

**Authors:** Peter H. Adler, Alparslan Yildirim, Zuhal Onder, G. Taskin Tasci, Onder Duzlu, M. Ozkan Arslan, Arif Ciloglu, Baris Sari, Nilgun Parmaksizoglu, Abdullah Inci

**Affiliations:** 1 Department of Agricultural and Environmental Sciences, 130 McGinty Court, Clemson University, Clemson, SC 29634-0310 USA; 2 Department of Parasitology, Faculty of Veterinary Medicine, Erciyes University, Kayseri, Turkey; 3 Department of Parasitology, Faculty of Veterinary Medicine, Kafkas University, Kars, Turkey; 4 Department of Microbiology, Faculty of Medicine, Kafkas University, Kars, Turkey

**Keywords:** Caucasus Mountains, nonrandom breakage, polytene chromosomes, sex linkage

## Abstract

An extreme example of nonrandom rearrangements, especially inversion breaks, is described in the polytene chromosomes of the black fly *Simulium bergi* Rubtsov, 1956 from Armenia and Turkey. A total of 48 rearrangements was discovered, relative to the standard banding sequence for the subgenus *Simulium* Latreille, 1802. One rearrangement, an inversion (*IIS-C*) in the short arm of the second chromosome, was fixed. Six (12.5%) of the rearrangements were autosomal polymorphisms, and the remaining 41 (85.4%) were sex linked. More than 40 X- and Y-linked rearrangements, predominantly inversions, were clustered in the long arm of the second chromosome (IIL), representing about 15% of the total complement. The pattern conforms to a nonrandom model of chromosome breakage, perhaps associated with an underlying molecular mechanism.

## Introduction

The sex chromosomes of the Simuliidae have commanded attention because of their suggested role in driving speciation (Procunier 1989, Rothfels 1989, Conflitti et al. 2015), a role also noted in other groups, such as the Drosophilidae (Presgraves 2008). Although cytologically undifferentiated sex chromosomes (X_0_Y_0_) are frequent in the Simuliidae, the X and Y of most species in the family are identified by sex-linked rearrangements easily visible in the polytene complement, most commonly inversions, but also heterobands, nucleolus organizer expression, and other phenomena (Post 1982, Procunier 1982a).

Unlike the separate heteromorphic X and Y chromosomes of organisms such as *Anopheles* Meigen, 1818 mosquitoes and *Drosophila* Fallén, 1823, any one of the three chromosomes (I, II, or III) functions as both the X and the Y chromosome in the Simuliidae. The simuliid sex-chromosome system, therefore, is more similar to that in the closely related family Chironomidae in which the sex chromosomes are generally undifferentiated, although males are sometimes distinguished by rearrangements, such as inversions, that mark the Y chromosome (Martin 1962, Newman 1977). Often, the linkage of rearrangements to sex in the Simuliidae is not complete, resulting in partial sex linkage (Rothfels 1980). Within species of the Simuliidae, sex-chromosome polymorphism is common but nearly always confined to a single chromosome (Adler et al. 2010). These sex-linked structural phenomena are typically paracentric in the long arm (L) or short arm (S), but also can be pericentric (Bedo 1977). Different sex chromosomes (e.g., I versus II) typically signal the presence of separate species (Bedo 1975, Brockhouse 1985). Sex-linked chromosomal rearrangements that produce heterozygosity suppress crossing over and the accompanying risk of breaking up adaptive complexes of sex-determination genes (Rothfels 1980, Post 1982).

Inversions often build on one another to produce elaborate sex chromosomes in a particular arm of the Simuliidae (Rothfels 1980). Even in species or groups of species in which the sex chromosomes are cytologically undifferentiated, the autosomal and fixed inversions tend to be concentrated in a few arms (e.g., Post et al. 2007, Adler et al. 2015).

Simulium (Simulium) bergi Rubtsov, 1956, a black fly in the *Simulium venustum* species group (Adler and Crosskey 2016), was described from the Lesser Caucasus of southern Georgia (Rubtsov 1956) and later discovered in Armenia (Terteryan 1968). It was long considered a Caucasian endemic (Chubareva and Petrova 2008) until its discovery in Ankara Province of Turkey (Crosskey and Zwick 2007). *Simulium bergi*, nonetheless, remains a geographically restricted, little-collected species; before our study, it had been recorded from only three sites.

General features of the karyotype of *Simulium bergi* from Armenia have been provided, such as the lengths of the polytene chromosomes (Chubareva and Petrova 1979). Photographs of the metaphase and the entire polytene complements and the polytene centromere regions also have been presented (Chubareva et al. 2003, Chubareva and Petrova 2008). We use the polytene chromosomes to explore the evolutionary relationships and cytogenetic structure of *Simulium bergi* at two sites: one in the Armenian Caucasus and one in eastern Turkey just beyond the western margin of the Lesser Caucasus. In particular, we examine the unique sex-chromosome system of *Simulium bergi*, which involves a large number of X- and Y-linked rearrangements in a restricted region of the polytene complement, to argue in favor of a nonrandom model of chromosomal reorganization.

## Material and methods

Larvae (penultimate and ultimate instars) were collected with forceps primarily from trailing vegetation in one stream each in Armenia and Turkey (Table 1) and fixed in 1:3 acetic ethanol (modified Carnoy’s solution). Our Armenian sample of 8 larvae was collected about 38 km south of the type locality of *Simulium bergi* in Tambovka, Akhalkalaki District, Georgia. Our Turkish sample of 30 larvae was collected about 105 km southwest of the type locality. The two sampling sites were less than 90 km apart. Larvae were identified morphologically using the keys and descriptions of Rubtsov (1956) and Terteryan (1968); identifications were confirmed chromosomally using the photomap of the complement presented by Chubareva and Petrova (2008).

**Table 1.** Collection sites for larvae of *Simulium bergi* used in chromosomal analyses.

Country	Location	Latitude Longitude	Elevation (m asl)	Collection Date	Females: Males
ARMENIA	Shirak Province, Saragyugh	41°08.51'N, 43°50.05'E	ca. 2150	14 June 2002	5:3
TURKEY	Kars Province, Bogatepe	40°48.37'N, 42°53.37'E	ca. 2200	07 June 2015	13:17

The posterior portion of each larval abdomen was removed and processed for Feulgen staining, following procedures of Rothfels and Dunbar (1953), but using 5N HCl at room temperature (Charalambous et al. 1996). One gonad and both salivary glands containing nuclei with the stained polytene chromosomes were dissected out with fine needles, placed in a drop of 50% acetic acid, and spread under a coverslip, with thumb pressure. Larval gender was determined by gonadal shape—slender and elongated in females and rounded in males—and confirmed cytologically by absence (females) or presence (males) of meiotic clusters.

The chromosomal banding sequences of all stained larvae were compared with maps of the standard reference sequence for the subgenus *Simulium*. For this comparison, we used the standard maps of Rothfels et al. (1978) for the IS, IL, IIL, and IIIS arms and of Adler et al. (2016) for the IIS and IIIL arms. Newly discovered inversions were numbered in order of discovery, following the last numbered inversion of Rothfels et al. (1978) for IS and IIIL and of Huang et al. (2011) for IIL. Heterobands (hb)—thickened bands (with enhanced DNA content) relative to the corresponding bands in the standard sequence—and thicker blocks of condensed chromatin, or heterochromatin (hc), not ascribable to a visible band were named for the section in which they occurred. Fixed rearrangements are italicized; all other rearrangements are not. Only chromosome arms with polymorphic rearrangements are shown in our figures (i.e., IS, IIL, and IIIL); other arms are identical to the sequences for the *Simulium venustum* group, including the fixed sequence in IIS, previously presented by Rothfels et al. (1978) and Chubareva and Petrova (2008). Thus, chromosomes IS, IIL, and IIIL of larvae collected in Turkey were photographed under oil immersion on an Olympus BX40 compound microscope. Chromosomal maps were constructed by scanning photographic negatives, with a 9000F Mark II CanoScan, and importing the images into Adobe® PhotoShop® Elements 8. All chromosomal rearrangements then were marked on the photographic maps to indicate their precise locations and breakpoints.

We identified the sex chromosome of *Simulium bergi*, based on a preponderance of one or more chromosomal rearrangements in one sex. We then followed the banding sequence of the homologue with the sex-linked rearrangement(s) to determine if additional rearrangements were on the same (cis) or a different (trans) homologue. In the heterogametic sex—the male (XY)—determination of linkage was complicated by heterozygosity. Thus, twisting and overlapping homologues sometimes could not be followed adequately to determine if two rearrangements were cis or trans; in these cases, X or Y linkage could not be resolved. Because females were the homogametic sex (XX), we inferred that any rearrangement in the IIL arm of females was X linked.

Stained and unstained portions of two Armenian larvae (and two pupae) and some larvae from Turkey were transferred to 80% ethanol and deposited in the Clemson University Arthropod Collection, South Carolina, USA, along with all photographic negatives of chromosomes. The majority of Armenian and Turkish larvae were placed in the Department of Parasitology, Erciyes University, Turkey, for future molecular analysis.

## Results

**Figure 1.**
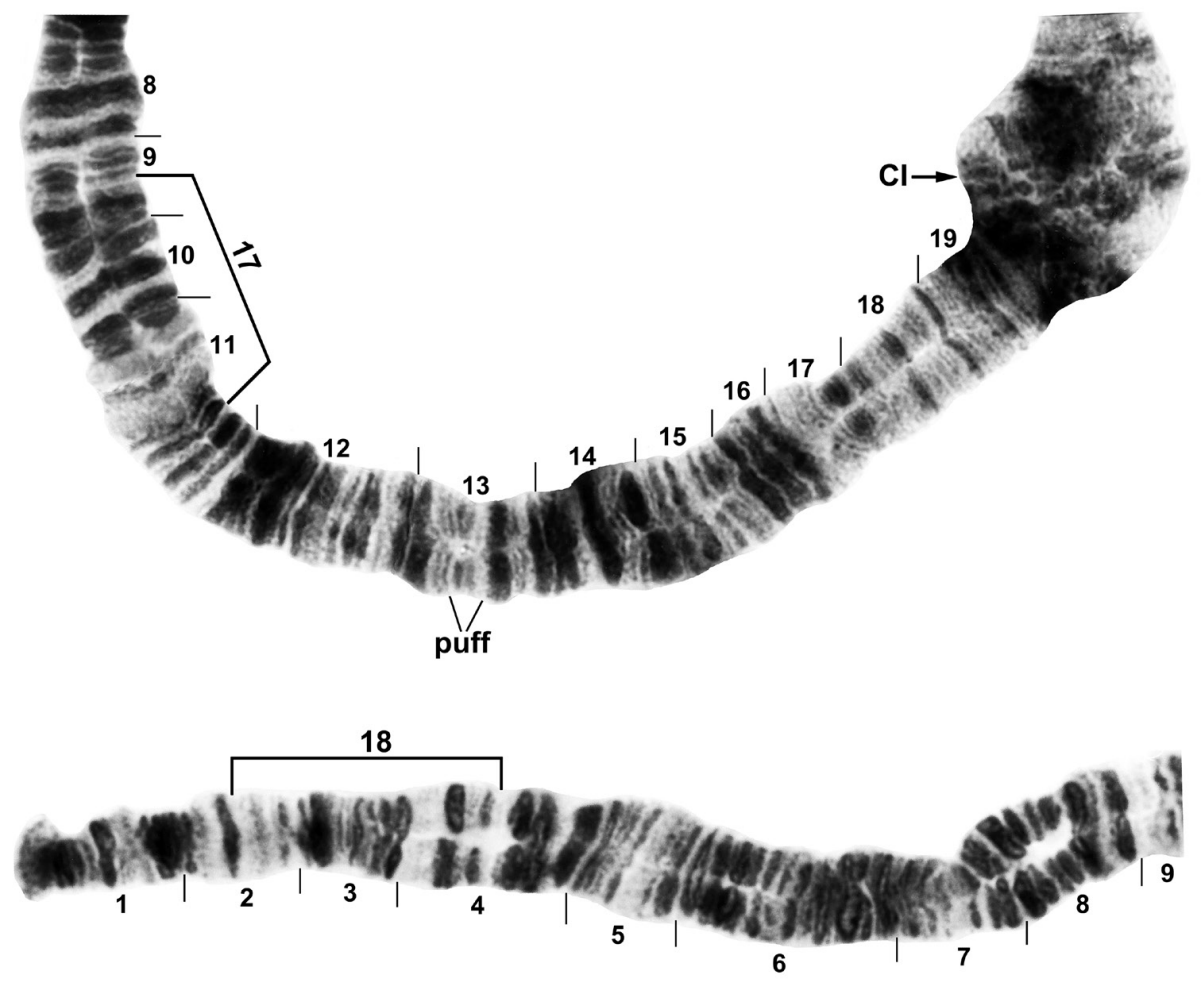
Chromosome arm IS of *Simulium bergi* (male larva), representing the *Simulium* subgeneric standard. Limits of two autosomal inversions and a puff are indicated with brackets on the standard sequence. CI = centromere of chromosome I.

**Figure 2.**
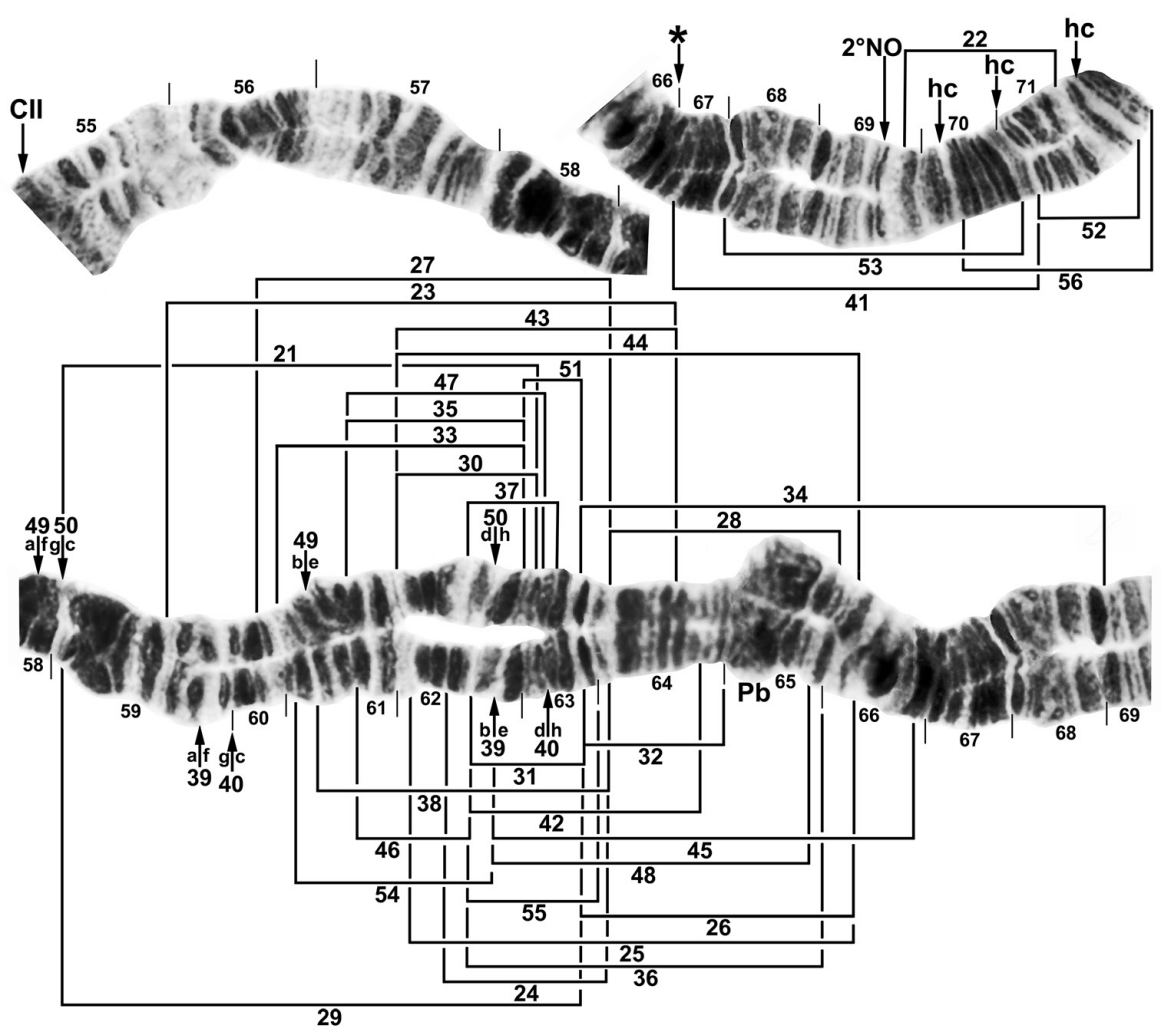
Composite map of chromosome arm IIL of *Simulium bergi* (female larva), representing the *Simulium* subgeneric standard. Breakpoints of sex-linked inversions are indicated with brackets or arrows. Ordering the two independent sets of chromosome fragments indicated by the letters “a” through “h” will produce the inverted sequence for IIL-39,40 and IIL-49,50. CII = centromere of chromosome II, hc = insertion point for heterochromatic block, Pb = parabalbiani, 2ºNO = location of secondary nucleolar organizer, * = insertion point for 7 additional bands (only when IIL-41 is present).

**Figure 3.**
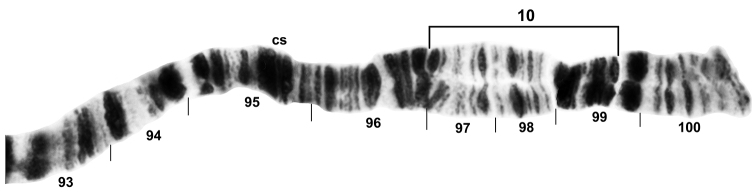
Distal half of chromosome IIIL of *Simulium bergi* (female larva), representing the *Simulium* subgeneric standard. Limits of autosomal inversion IIIL-10 are indicated with a bracket on the standard sequence; cs = cup and saucer marker.

**General features.** The banding sequences of all 38 larvae (18 females, 20 males) of *Simulium bergi* were analyzed completely. The general features of the polytene complement conformed to the photograph by Chubareva and Petrova (2008). All larvae had the typical *n* = 3 haploid complement, with submetacentric chromosomes, and the lengths expressed as I > II ≈ III. Homologues were tightly paired (Figs 1–3). A chromocenter and supernumerary chromosomes were absent. Ectopic pairing of centromeres occurred in 0–20% of the nuclei of each larva. Centromere bands were diffuse and within expanded regions; the CI region (Fig. 1) was the most expanded, followed by the CII and then the CIII regions. The nucleolar organizer was in the standard subgeneric position in the base of IIIL at the junction of sections 87 and 88.

**Fixed inversions.** The fundamental banding sequence common to all larvae was derived from the standard subgeneric sequence by a single fixed inversion, *IIS-C* (*sensu* Rothfels et al. 1978), which reversed the ring of Balbiani and the “bulges” marker. The IIS sequence, therefore, was identical to that in figure 11 of Rothfels et al. (1978) and figure 210 of Chubareva and Petrova (2008).

**Autosomal polymorphisms.** Six autosomal polymorphisms were discovered (Table 2): IS-17, IS-18, IIIL-10, one puffed band in IS (Figs 1, 3), and two heterobands in IL. All six of these rearrangements occurred only once each and only in the heterozygous condition, except IIIL-10 (Fig. 3), which was found in 29% of all larvae, including homozygously in one Armenian larva. IIIL-10, was shared between Armenian and Turkish populations, although its frequency was significantly greater in Armenia (χ^2^ = 11.9, df = 1, p = 0.001).

**Table 2.** Frequency of homologues with autosomal rearrangements in two populations of *Simulium bergi*.

	ARMENIA	TURKEY
Larvae (*n*)	8	30
Homologues (n)	16	60
IS-17^†^	0.00	0.02
IS-18	0.06	0.00
IS-puff(13)	0.06	0.00
IL-hb26	0.06	0.00
IL-hb(telomere)	0.00	0.02
IIIL-10	0.44	0.08

† Left column represents autosomal rearrangements, all of which are indicated on Figs 1, 3; frequencies are based on a maximum of 1.00.

**Figure 4.**
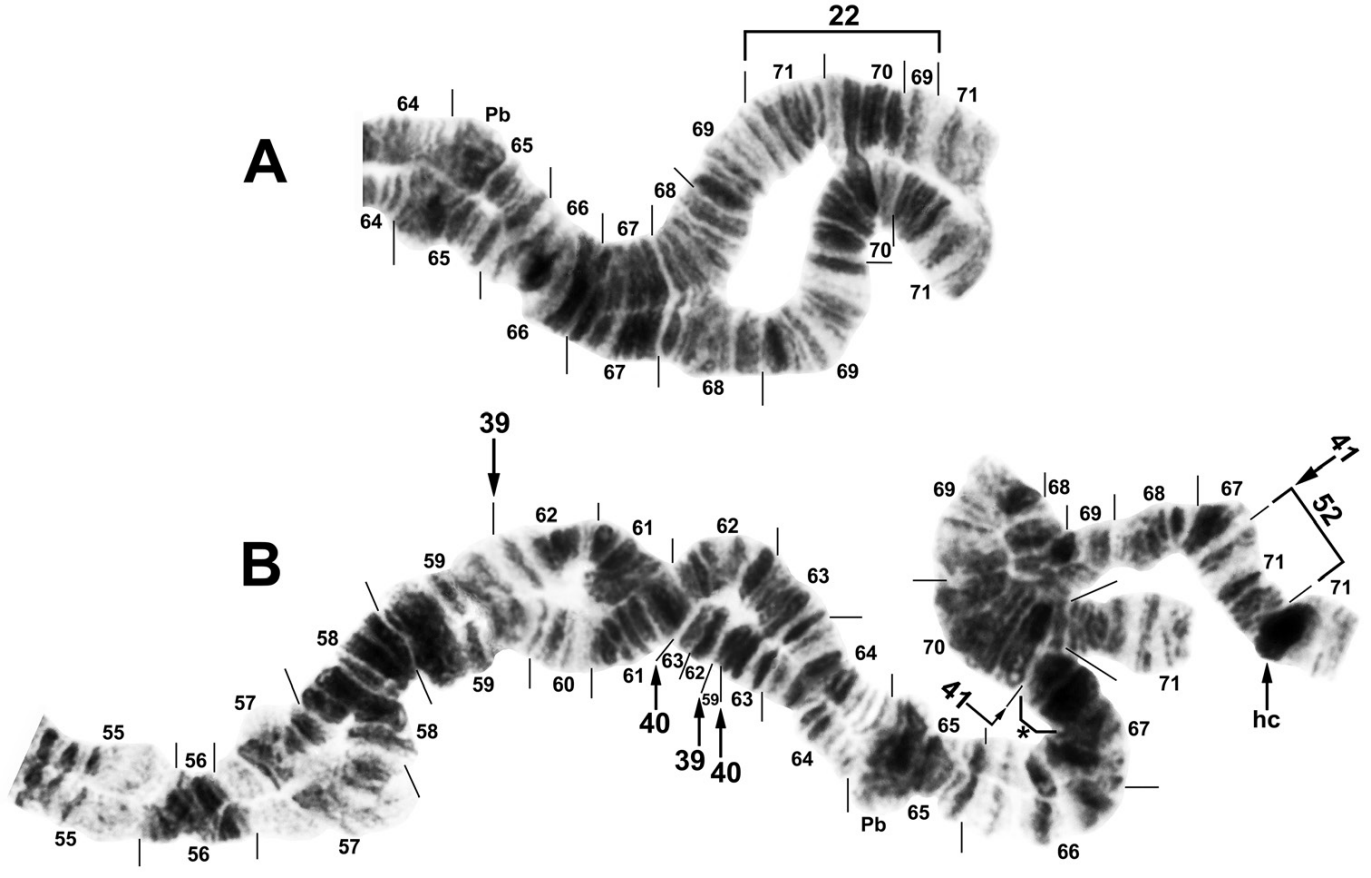
Chromosome arm IIL of male larvae of *Simulium bergi*. **A** Distal half of chromosome, showing heterozygous expression of the common Y-linked inversion IIL-22 **B** Complex sex-chromosome configuration, showing one homologue with IIL-39,40 and the other with IIL-41,52+hc71+7 extra bands; hc = heterochromatic block, Pb = parabalbiani, * = 7 additional bands inserted in one homologue.

**Figure 5.**
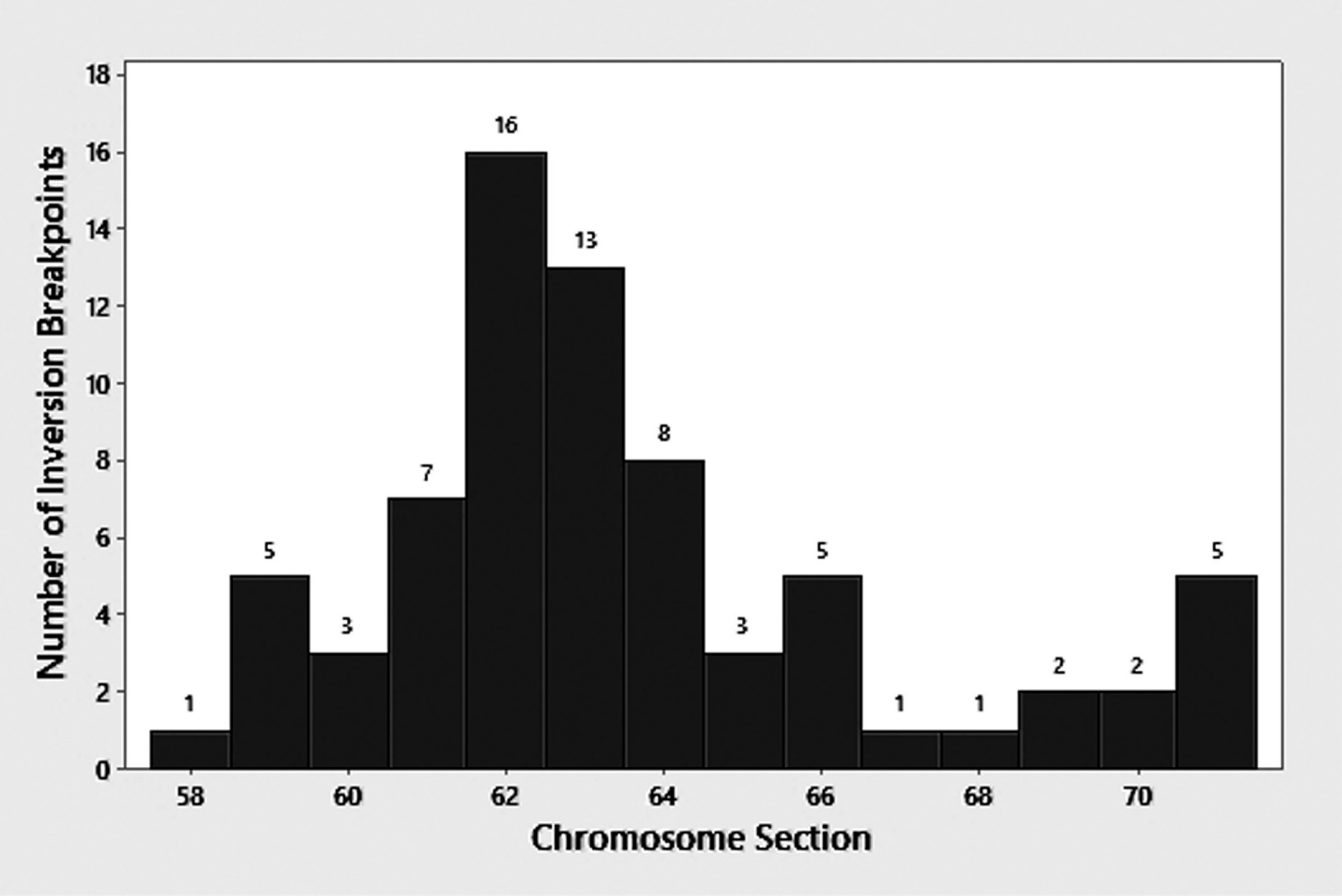
Distribution of breakpoints of 36 sex-linked inversions in the IIL arm of *Simulium bergi*. Breakpoints are plotted according to section number of the standard banding sequence for the subgenus *Simulium*. Breakpoints falling at the junction of two sections are tallied for the distalmost of the two sections.

**Sex chromosomes.** IIL was inferred as the sex arm in the Turkish population (Table 3), based on inversion IIL-22 (Figs 2, 4A), which appeared exclusively in the heterozygous condition in 76.5% of the 17 males and in none of the 13 females; no IIL-22 homozygotes were found. We tentatively consider IIL as the sex arm in the Armenian population where 1 of the 3 Armenian males had IIL-22, although a larger sample is needed to test the hypothesis of Y linkage. Our combined sample of 38 larvae included 41 rearrangements in IIL (Fig. 2). All 38 larvae were heterozygous for at least one rearrangement in IIL. By following the homologue with IIL-22, we established that this inversion was on the same homologue as four other inversions (IIL-23, IIL-34, IIL-43, and IIL-56); these inversions, therefore, also were linked to the Y chromosome. More than 20 rearrangements were linked to the X chromosome. An X chromosome (X_0_) with no rearrangements occurred in both Armenia and Turkey. Of 41 rearrangements in IIL, 16 (all in males) could not be determined as linked to either the X or the Y, including one larva with the most complex set of rearrangements (IIL-39,40 on one homologue and IIL-41,52+hc71+7 extra bands on the other homologue; Fig. 4B). Four IIL rearrangements (IIL-22, IIL-23, IIL-30, and IIL-44) were shared between Armenia and Turkey. The concentration of 36 inversions in IIL involved some pairs, such as IIL-35 and IIL-47, that differed by only one or two visible bands. The distribution of breakpoints for the sex-linked inversions followed a bimodal distribution, with a nearly normally distributed central cluster and a smaller subterminal cluster (Fig. 5).

**Table 3.** Number of larvae with each sex-linked rearrangement in two populations of *Simulium bergi*.

	Armenia	Turkey	X or Y Linked^†^
Females: Males	5:3	13:17	–
IIL-21^‡^		1	
IIL-22	1	13	Y
IIL-23	1	3	X, Y^§^
IIL-24		2	
IIL-25		2	X
IIL-26		2	X
IIL-27		1	
IIL-28		3	
IIL-29		2	X
IIL-30		4	X
IIL-31		2	
IIL-32		1	
IIL-33		1	X
IIL-34		2	Y
IIL-35		1	X
IIL-36		1	X
IIL-37		1	X
IIL-38		1	
IIL-39		2	
IIL-40		1	
IIL-41		1	
IIL-42		1	X
IIL-43		3	X, Y
IIL-44	3	1	X
IIL-45		1	
IIL-46		1	
IIL-47		1	
IIL-48		1	
IIL-49		1	X
IIL-50		1	X
IIL hc71		1	
IIL extra bands^|^		1	
IIL-51		1	X
IIL-52	1		Y
IIL-53	1		X
IIL-54	1		X
IIL-55	1		X
IIL-56	2		Y
IIL hc70	1		X
IIL hc70/71	1		X
IIL 2ºNO	1		X

† An empty cell indicates that X or Y linkage of the rearrangement could not be determined.

‡ Left column represents sex-linked chromosomal rearrangements in the IIL arm, all of which are indicated on Figs 2, 4; frequencies are based on a maximum of 1.00.

§ IIL-23 was in cis conformation with IIL-22 in Armenia, where we tentatively consider IIL-22 to be Y linked, based on only 3 males available for study. IIL-23 was associated with the X chromosome in Turkey.

| Seven extra bands appeared in one homologue at the junction of sections 66/70 created by IIL-41; the same homologue had IIL-52 and IIL hc71, and the opposite homologue had IIL-39,40 (Fig. 4B).

## Discussion

**Chromosomal insights into taxonomy.** We consider our Armenian and Turkish populations of *Simulium bergi* conspecific, based on shared chromosomal characters, *viz.*, the entire fixed banding sequence, autosomal polymorphism IIIL-10, and four sex-linked inversions. Rearrangements unique to Armenia or Turkey probably reflect, in large part, small sample sizes. We would not expect restricted gene flow, given the flight capabilities of simuliids (Adler et al. 2005) and the short distance (< ca. 100 km), availability of appropriate breeding habitats, and similar elevations between our two sampling sites and between either of our two sampling sites and the type locality in southern Georgia. These three sites also are in the same ecoregion, the Eastern Anatolian Montane Steppe (World Wildlife Fund 2015). We, therefore, suggest that our populations are conspecific with the type specimen. Conspecificity with more distant populations is unknown. The only insight comes from a photograph of the total polytene complement of *Simulium bergi* collected from the Argichi River about 165 km southeast of our Armenian site (Chubareva and Petrova 2008). It shows the standard subgeneric banding sequence in chromosomes I and III and the *C* sequence in IIS. IIL is standard, although sections 68–69 (= sections 26–27 on the map of Chubareva and Petrova 2008) appear knotted and might have either an extra block of heterochromatin expressed heterozygously or a small inversion that cannot be interpreted.

The presence of *IIS-C* chromosomally confirms the original (Rubtsov 1956) morphological placement of *Simulium bergi* in the *Simulium venustum* species group. Of the 18 nominal species in the *Simulium venustum* group analyzed chromosomally (Adler and Crosskey 2015), *Simulium bergi* is the least differentiated—only one inversion (*IIS-C*) removed from the subgeneric standard. However, one other analyzed member of the group, *Simulium paramorsitans* Rubtsov, 1956, also has a fixed banding sequence (Adler et al. 1999) identical to that of *Simulium bergi*. The two species are, therefore, homosequential (*sensu* Carson et al. 1967); that is, they have the same fixed chromosomal banding sequence but differ morphologically, especially in their larval head patterns, as shown by Rubtsov (1956). Although their fixed sequences are identical, *Simulium paramorsitans* and *Simulium bergi* are at opposite extremes in the differentiation of their sex chromosomes: undifferentiated in the former (Adler unpublished) and highly diverse in the latter. Other Palearctic members of the *Simulium venustum* group, such as *Simulium longipalpe* Beltyukova, 1955 (formerly *Simulium curvistylus* Rubtsov, 1957), *Simulium morsitans* Edwards, 1915, *Simulium posticatum* Meigen, 1838, and *Simulium rubtzovi* Smart, 1945 (Adler et al. 1999), show only slight fixed chromosomal differentiation from *Simulium bergi*. The absence of any shared chromosomal rearrangement, other than *IIS-C*, with other members of the *Simulium venustum* group precludes determination of the species most closely related to *Simulium bergi*.

**Chromosomal fragility in the sex arm.** *Simulium bergi* represents the most extreme known case of sex-chromosome differentiation in the Simuliidae, with 41 sex-linked rearrangements discovered on IIL (i.e., the sex arm) among 38 larvae. IIL distal to section 58, thus, is an area of rearrangement hotspots, particularly in the central region of the arm. This area of fragility includes sets of mimic inversions, two or more sequence reversals that resemble one another, differing by as little as one band (Rothfels 1989).

Widespread species can exhibit a significant cumulative degree of sex-chromosome polymorphism over their entire geographical distribution, with inversions often replacing one another across the distribution (e.g., *Simulium vittatum*, Zetterstedt 1838; Rothfels 1980). Typically, however, the number of sex-linked rearrangements is more limited within a population, often to a single example, linked either to the X or the Y (Adler et al. 2016). Yet, examples of multiple sex-linked rearrangements in a single population are not uncommon. Two cytoforms (‘A’ and ‘D’), probably cryptic species, of *Simulium colombaschense* (Scopoli, 1780), each exhibits sex-chromosome polymorphism within a population at one river site; ‘A’ has 4 X-linked and 1 Y-linked inversions at a single site, and ‘D’ has 4 X-linked and 4 Y-linked inversions (Adler et al. 2016). *Simulium conundrum* Adler, Currie & Wood, 2004 (formerly *Simulium tuberosum* ‘FGH’) at a single site in Newfoundland has 4 Y-linked inversions but no X-linked inversions (McCreadie et al. 1995). In contrast, the number of rearrangements, per geographic site, linked to sex in *Simulium bergi* is extraordinary. A single Y-linked inversion (IIL-22), nonetheless, forms the backbone of the sex-chromosome system, occurring in 76% of Turkish and 33% of Armenian male larvae. The X chromosome is indiscriminate in its sex-linked rearrangements; none of the 20 or more rearrangements is represented in more than 4 (22%) of the 18 total female larvae.

In species with multiple rearrangements linked to sex, the sex-differential region tends to become progressively enlarged (Rothfels 1980). In the *Simulium ochraceum* species complex from Central America, for example, 2 X chromosomes and 6 Y chromosomes are found in populations of *Simulium ochraceum* ‘A’. Sex linkage in this species involves not only inversions along the entire IIL arm, but also a supernumerary band polymorphism (Hirai et. al. 1994). By contrast, *Cnephia dacotensis* (Dyar & Shannon, 1927) is one of the few species that shows sex-differential regions spanning the centromere region, and is unique in that the sex chromosomes involve only polymorphic bands in chromosome I (Procunier 1975, 1982b). *Simulium bergi* shows a concentration of inversions between sections 60 and 65, with additional, albeit fewer, inversions breaking beyond this segment.

At least two inversions, IIL-23 and IIL-43, were linked predominantly to the X, but in one larva each, they were linked to the Y. Sex exceptions in the Simuliidae are frequent (e.g., Rothfels & Featherston 1981). They have been considered ancestral relicts, the result of crossing over, or products of transposable element excision (Rothfels 1980, Brockhouse 1985, Bedo 1989).

The concentration of inversions in particular areas of the macrogenome, such as the IIL arm of *Simulium bergi*, emphasizes that inversions are not random. Inversion concentrations have been known and easily visualized for decades in the polytene chromosomes of dipterans (e.g., Novitski 1946, Rothfels and Fairlie 1957) including the Simuliidae (Landau 1962). Yet, a random model was invoked for many years to explain two-break chromosomal rearrangements, such as inversions, at least in mammalian systems (Nadeau and Taylor 1984). Nonrandom models to explain inversion clusters have been proposed, such as the stress-in-pairing model (Rothfels and Fairlie 1957) and fragile breakage model (Bailey et al. 2004), the latter based on mammalian chromosomes that do not offer the polytene benefit of detailed visualization. Repair of breaks depends on multiple factors, such as chromosome position (Lee et al. 2016). Breakage in chironomid midges has been hypothesized to occur preferentially in areas of the complement containing repetitive blocks of DNA (Bovero et al. 2002). In *Anopheles* mosquitoes, the X chromosome, which has accumulated inversions about three times more rapidly than have the autosomes, is associated with high densities of transposable elements and satellites, suggesting a possible mechanism for the origin of inversions (Sharakhov et al. 2016). Transposable elements have been invoked to explain the presence of multiple sex-determining regions in species of the Simuliidae, although direct evidence is still wanting and alternative models have been considered (Procunier 1982b, Bedo 1984, Brockhouse 1985). In support of a nonrandom model is the association of high G+C regions with areas of high-frequency breakage (Webber and Ponting 2005). Also relevant to general genome organization is the finding that AT-rich (heterochromatic) polytene bands of the *Simulium vittatum* complex are randomly dispersed throughout the complement (Procunier and Smith 1993).

We draw attention not only to clustered inversions in the distal three-quarters of the IIL sex arm, but also to the clustering of other rearrangements. Five structural phenomena, other than inversions, such as heterochromatic blocks, are found in the sex arm (15% of the polytene complement) compared with three in the autosomal portion (85% of the complement). If the pattern is consistent, it suggests that models accounting for increased inversion frequency also should accommodate the increased frequency of nonbreak-type rearrangements, such as the addition of heterochromatin. The functions of heterochromatin are varied and include suppression of recombination (Grewal and Jia 2007), potentially contributing to the maintenance of blocks of genes related to sex determination.

What is not known is whether a particular area of the complement—the IIL arm of *Simulium bergi*, for example—is more susceptible to breakage or if the breaks that occur are more likely to persist through subsequent generations, or if both phenomena play a role. Also unknown is whether a visualized break shared by two or more inversions is equivalent at the level of the base pairs. This question can be addressed through molecular characterization of the distal and proximal breakpoint sequences (Sharakhov et al. 2006). *Simulium bergi* offers an exceptional case for exploring the molecular nature of chromosomal breaks and other rearrangements related to the sex chromosomes, while affording a physical map of the phenomenon.
